# The feasibility of the enhancing medicines self-management for community dwelling people living with dementia and family carers (MAGNET) psychosocial intervention: protocol for a non-randomised feasibility study

**DOI:** 10.1186/s40814-025-01739-w

**Published:** 2025-12-16

**Authors:** Lubena Mirza, John L. O’Dwyer, Mariam Fargin, Beth Fylan, Zoe Edwards, Justine Tomlinson, Mohammed Akhlak Rauf, Catherine Quinn, Catherine Powell

**Affiliations:** 1 NIHR Yorkshire and Humber Patient Safety Translational Research Centre, Bradford Institute for Health Research, Bradford, UK; 2 Wolfson Centre for Applied Health Research, Bradford, United Kingdom; 3 Academic Unit of Health Economics, Leeds Institute of Health Sciences, University of Leeds, Worsley Building, Leeds, LS2 9NL UK; 4 School of Pharmacy and Medical Sciences, University of Bradford, Bradford, UK; 5 Affinity Care PCN, Bradford, United Kingdom; 6 Meri Yaadian, Bradford, United Kingdom; 7 Centre for Applied Dementia Studies, University of Bradford, Bradford, UK

**Keywords:** People with dementia, Family carers, Psychosocial interventions, Resilience, Non-randomised feasibility study

## Abstract

**Background:**

Medicines management for people with dementia is a global health priority. Cognitive difficulties, such as memory problems, can impact safe and effective medicines management. With appropriate support, people with mild–moderate dementia can self-manage medicines, with and without family. People with dementia can enhance medicines safety by building resilience into their medicines management system through their ability to respond, learn, monitor and anticipate. Medicines self-management interventions have more often focused on adherence as opposed to other experiences, such as knowledge of medicines, though psychosocial interventions have addressed other components of medicines self-management. Co-design approaches draw on the person's experience. A co-designed resilience-enhancing psychosocial intervention for medicines self-management for community-dwelling people living with dementia and with or without family carers (MAGNET) was developed. This protocol describes a study aiming to assess the feasibility and acceptability of the MAGNET intervention.

**Methods:**

Study objectives are to assess the feasibility and acceptability of implementing the MAGNET intervention in preparation for a randomised controlled trial; to develop materials for estimating the effectiveness and cost-effectiveness of the intervention in a randomised controlled trial, and to assess the feasibility and acceptability of collecting data using these materials. This is a non-randomised feasibility study. Seventy-two people living with dementia and their family carers will be recruited to the study and receive the MAGNET intervention. The intervention involves an 8-week community-based medicines self-management programme involving a ‘Managing My Medicines’ guide for people living with dementia and carers, and a medicines self-management professional's guide to support implementation by trained professionals. Methods of evaluation include questionnaires and interviews with people living with dementia, family carers and professionals. Intervention delivery will be observed. Quantitative and qualitative data will be completed at baseline, end month 1, end month 2, and post intervention. The evaluation will be underpinned by the Consolidated Framework for Implementation Research (CFIR).

**Discussion:**

This study will indicate whether the MAGNET intervention is feasible and acceptable. If the study findings support feasibility and acceptability, then we will seek further funding for the development and implementation of a randomised controlled trial.

**Trial registration:**

ISRCTN 15712227.

## Background

Medicines management for people living with dementia is a global health priority. Approximately 50 million people worldwide are living with dementia [1]. Polypharmacy and comorbidities have a higher prevalence in those with dementia compared to those without. The complexity of medicines management for people living with dementia can be impacted by cognitive difficulties, such as memory problems; medication-related factors, such as medication error; social factors, such as family carer support; and communication and health literacy, such as the ability to understand a complex regimen [2]. There is a two-way relationship between health literacy and cognitive ability [3]. Medicines management issues include limited holistic approaches and communication between professionals, and family carers lacking guidance on medication management [4]. A scoping review underscored the significant challenges to medication management information needs faced by people living with dementia and their family carers [5]. The most frequently reported challenge identified in the review was missing, limited, or not useful critical medicine information [6–28]. There was a need for simpler tailored relevant information [18, 29].

A self-management approach has the potential to support safe and effective medicines management for people living with dementia and family carers. Self-management typically involves drawing on one’s own knowledge and skills; goal setting; monitoring behaviour and evaluation [30]. Family carers also need to be considered in self-management interventions as they are frequently involved in dementia care in the home [31]. Benefits of a self-management approach include enhancing quality of life, self-efficacy, knowledge, skills to manage illness and reducing stress [32]. Such improvements have been found for those living with mild–moderate dementia [31, 33]. Moreover, patients can enhance safety by building resilience into their medicines management system [34]. A resilient healthcare (Safety II) approach emphasises how the flexibility and adaptability of healthcare systems result in safer outcomes in the face of fluctuations in normal system operations and disruption. Resilience abilities can include: ability to respond, such as being able to respond to changes; ability to monitor, awareness of what to look out for; ability to learn, awareness of what has happened and being able to learn from this; and ability to anticipate, such as knowing what to expect [35]. Patients and their families have a key role in this process for medicines management [34].

We conducted a systematic review to identify the existing evidence about interventions to support medicines self-management for people living with dementia (or cognitive impairment), and family carers in medicines self-management [36]. Definitions of medicines self-management more often emphasise adherence, whereas patient perspectives are more evident in studies of health literacy [37]. The patient’s experience of medicines self-management can include checking for errors, seeking support, adherence, knowledge, supply management and monitoring effects [34, 38–40]. We further checked our understanding of the definition through discussions with a patient public involvement (PPI) group that was identified and invited through an existing university PPI network. An online meeting was held, followed by a phone meeting, and correspondence via email. Further information about the PPI group’s involvement can be seen in the systematic review paper [36]. We screened articles for medicine self-management components and resilience capabilities. Key findings from the systematic review indicated that people living with dementia or cognitive impairment and their family carers need support with self-management of medicines. Interventions primarily focused on improving adherence through providing reminders via devices and pictures [41], rather than addressing other key person-centred experiences of medicines self-management such as re-ordering, checking supplies and error resolution, seeking support about medicines from healthcare professionals, monitoring effects and side-effects, and having knowledge about medicines. Medicines self-management interventions for people living with dementia and family carers that aim to enhance resilience were limited. However, psychosocial interventions showed some promise in addressing a broader conception of medicines self- management, had stronger theoretical underpinnings in social cognitive and self-efficacy theories, and enhanced a greater number of resilience outcomes [42, 43]. All interventions lacked patient public involvement (PPI) in their development, a co-design approach, and none were UK-based. Not involving end users in intervention development through co-design will limit the effectiveness in addressing real needs and patient priorities, that go beyond adherence [36].

Thus, there is a need for a co-designed theoretically informed psychosocial intervention that enhances resilience, and supports not only adherence to medicine regimes, but more aspects of self-managing medicines when living with dementia, including re-ordering, checking supplies and error resolution, seeking support from health care professionals with medicines, monitoring effects and side effects, and having knowledge about medicines.

In the first two phases of the MAGNET programme, using the theory of resilience in healthcare, we explored how people with mild-moderate dementia (with and without their family) self-managed their medicines. Using an Experience-Based Co-Design (EBCD) approach, we co-designed an intervention with people living with dementia, family carers, health, social care, and voluntary sector professionals who regularly support safe medicines self-management. EBCD is a participatory method, where patients, the public, and professionals involved in a service are brought together to co-design services [44]. Whilst this method is usually conducted in single organisations for service improvement purposes, EBCD has been adapted in several ways [45], including implementation across multiple sites and for research purposes [46]. The first and second phases of the MAGNET programme will be published in separate papers. In this third phase of the project, we will assess the feasibility and acceptability of the MAGNET intervention.

## Methods

Our key aim is to assess the feasibility and acceptability of the MAGNET intervention.

Our key objectives are to:

i) Assess the feasibility and acceptability of implementing the MAGNET intervention in preparation for a randomised controlled trial.
ii) Develop materials for estimating the effectiveness and cost-effectiveness of the intervention in a randomised controlled trial.
iii) Assess the feasibility and acceptability of collecting data using these materials.

If the results support feasibility and acceptability:

- We will use the findings to inform the development and implementation of a future randomised controlled trial, for which we will seek onward funding,
- Have a proposal for a randomised controlled trial if appropriate.

### Design

This study will use a non-randomised feasibility design and will follow the SPIRIT 2013 guidelines [47] and reporting template (Additional file 1). A non-randomised design is selected because we are concerned with the feasibility and acceptability of the MAGNET intervention, not measuring effectiveness. The non-randomised design will provide us with data about how the intervention could work within real-world settings. Moreover, time and resources are limited within a planned two-year multiphase programme involving exploring how people with mild-moderate dementia (with and without family carers) self-manage medicines, co-designing an intervention, and conducting a feasibility study. We recognise the limitations that a non-randomised study can introduce through for example selection bias and providing misleading results [48]. Hence, we aim to conduct a randomised controlled trial when measuring effectiveness should the intervention be proved feasible and acceptable following this feasibility study. A schedule for study procedures is provided in Table 1.

**Table 1:** Study procedures

Procedures	Visits					
	Screening	Baseline	Months			Post intervention
			Start 1	End 1	End 2	
Informed consent people living with dementia and family carers	X					
Informed consent of professionals	X					
Questionnaires with people with dementia and family carers		X		X	X	X
Interviews with people with dementia and family carers						X
Interviews with professionals						X
Observation of intervention delivery			X			
Intervention delivery (including staff training, guide delivery)		X	X			
Phone calls between professionals and people with dementia and family carers				X		

### Intervention

The MAGNET intervention is delivered over 8 weeks. Materials include: the MAGNET guide for professionals to implement the intervention, the MAGNET guide for people living with dementia and their family, a short video summary of the intervention a reminder postcard, and associated training for professionals. The training for professionals comprises two sessions of 45 min each, which can be delivered online or in person. Professionals will deliver the intervention, ‘Managing My Medicines’ Guide, to the person (and family carer if relevant) and facilitate the identification of key goals and actions related to medicines self-management. After 4 weeks, the professional will contact the person (and family carer if relevant) to provide further support as required. Due to the diverse population and services delivering the intervention, how long the professional will spend at each visit will vary. Some professionals will be delivering the intervention as an add-on to the usual care visit. We anticipate the first visit lasting 45 min, and the follow-up visit lasting 30 min.

### Recruitment

**Fig. 1:**
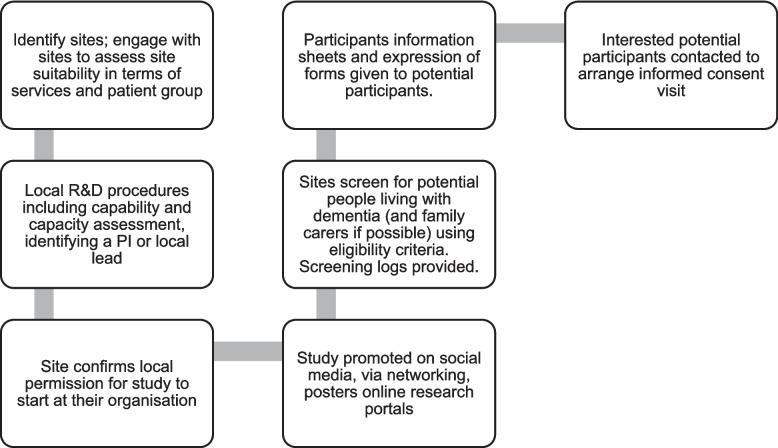
Recruitment process

Figure 1 below shows how sites and participants will be identified and recruited to the study.

The National Institute for Health and Care Research, Research Delivery Network (RDN) will send out expressions of interest to English NHS Trusts, to identify eligible sites. The research team will also identify relevant organisations through internet searches and networking, followed by in-person or online meetings to further engage with interested sites. Eligible sites will be those supporting people who are living with dementia, including mental health trusts, community health trusts, general medical practices (GPs), memory clinics, primary networks, local councils, community pharmacies, social enterprises, and research platforms.

The study team will liaise with recruitment sites for ideas and suggestions about how a diverse sample can be achieved. The participation of sites such as GPs, community health trusts, memory clinics, social enterprises will enable invitations for participation to reach a wider and more diverse population.

### Participants

We plan to recruit 72 participants who will be community-dwelling people living with dementia and family carers will also be invited to participate. Professionals will be recruited from the health, social care or voluntary sector. We will decide whether participants will be eligible to engage in the study on a rolling basis in a future randomised controlled trial when we consider the results of the feasibility study.

### Inclusion criteria

People living with dementia will be eligible to take part if they are.

- Living with mild-moderate dementia (as indicated by a Mini-Mental State Examination [49] score between 24 and 10 or a clinical diagnosis of mild-moderate dementia).
- Using two or more medicines.
- Living at home.
- Living alone or living with family carers.

Family carers will be:

- Relatives, friends or neighbours identified as involved in supporting the person with their medicines.
- Caring for the person with mild-moderate dementia (as indicated by a Mini-Mental State Examination [49] score between 24 and 10, or a clinical diagnosis of mild-moderate dementia), using two or more medicines, and living at home.
- May be living within or outside the household of the person with dementia.

Professionals will be.

- Health, social care or voluntary sector professionals who support people with dementia and family carers or researchers with relevant health, voluntary or social care background.

### Exclusion criteria

People with dementia will be excluded if they are any of the following:

- Using fewer than two medicines.
- Living in care homes.
- Live with moderate to advanced dementia.
- Lack capacity to consent.
- On the End-of-Life Pathway.
- Unable to communicate verbally.

Family carers will be excluded if they are either of the following:

- Not a relative, friend or neighbour identified as involved in care for the person in this study.
- The person they are caring for has advanced dementia, using fewer than two medicines, and/or not living at home.

Professionals will be excluded if they are any of the following:

- Have no involvement with patients with mild-moderate dementia living at home or are not researchers with a relevant health, voluntary or social care background.
- Have no involvement with patients with mild–moderate dementia, on two or more medicines or are not a researcher with a relevant health, voluntary or social care background.

### Sample size

We will recruit 72 people living with mild-moderate dementia and family. We predict that, with dropout, 60 recruited participants would yield a sufficient response rate to meet the study objectives. Health, social and voluntary sector professionals will be recruited across the three healthcare areas. The sample size of professionals is dependent on how many professionals are required at each organisation to deliver the intervention. We will liaise with sites directly to discuss capacity and capability at each organisation. We anticipate approximately 27 professionals will be recruited.

Given this is a non-randomised feasibility study, a formal sample size calculation is not needed [50]; however, statisticians at a clinical trials unit were consulted for advice. The sample size is based on the need to estimate the recruitment rate, and our knowledge of numbers available and recruitment method [51]. We aim to understand if we can consistently recruit 72 over a period of 6 weeks, four a week for 6 weeks in three sites. We will analyse drop-out rates for the 72 as the intervention is delivered. We predict that, with drop-out, 60 recruited patients would yield a sufficient response rate to meet the study objectives. The decision on 60 recruited participants (30 people with dementia/family carer dyads) was based on the general rule that at least 30 participants or greater are needed to estimate parameters [52, 53]. Should we apply for a randomised control trial, we will use data from the feasibility study to inform the parameters used for the estimation of the sample size.

### Control group

The study is a pretest post-test design. It will be a single-arm feasibility study and will not have a comparison group.

### Data collection

Qualitative and quantitative data will be collated. Data will be collected at baseline (preintervention), end month 1 and 2 (questionnaires) post intervention (follow up at month 3) (interviews and questionnaires). A total of 20 separate interviews will be conducted with people living with dementia and family carers. Reminders for data collection will be sent where relevant.

Qualitative and quantitative data will also be collected from professionals. Data will be collected at baseline (pre-intervention questionnaire involving the PCAT Pragmatic Context Assessment Tool [54], at the point of intervention delivery (observations), postintervention (interviews and questionnaires). Facilitators will complete a checklist reporting intervention use.

### Qualitative data collection

Interviews will be semi-structured. Interview schedules with a range of questions, probes and prompts will explore the patient experiences of the intervention components and the professionals’ experience. The schedule will be underpinned by the consolidated framework for implementation research (CFIR) [55, 56], social cognitive [57], self-efficacy [58], and resilience theory [35]. Participants will be asked about any processes that enhanced their experience with their medicines. Interviews will last approximately 45 min, will be audio recorded with participants’ written permission and will be transcribed verbatim.

### Observations

We will seek permission to conduct 20 observations of intervention delivery to understand how the toolkit is being executed in practice, and the culture within which the intervention is being implemented. The observation will be carried out in the home of the person with dementia, or a relevant community setting, according to the participant’s choice about where the intervention is being delivered. Focused and general observation data will be collected with a structured observation tool and field notes. The fieldnotes will be semi-structured with prompts, again aligned to the consolidated framework for implementation research (CFIR) [55, 56], social cognitive [57], self-efficacy [58, 59], and resilience theory [35], as well as findings from previous programme phases.

A potential limitation of conducting overt observation is the Hawthorne Effect by which individuals being observed may alter their behaviour because they are aware that they are being studied [60]. We will seek to minimise the impact by informing participants that we wish them to behave as normal. A maximum of two researchers will be present at any one time.

### Feasibility study outcomes

We will assess the recruitment strategy, capability and sample characteristics; data collection procedures and outcome measures; and intervention acceptability, study procedures, and preliminary responses to the intervention. We will determine the main outcome measure for a future randomised controlled trial. Feasibility study outcomes are highlighted in Table 2.

**Table 2:** Feasibility study outcomes

Recruitment strategy, capability and sample characteristics	
1	Number of people with dementia and family carers who are screened, eligible and consent
2	Recruitment of sites
3	Recruitment of facilitators to deliver the intervention
4	Recruitment strategy for people with dementia and family carers
5	Diversity of sample of people with dementia and family carers
6	Reasons for non-recruitment or attrition
7	Dropout rates and eligibility to inform sample size estimates for a future definitive trial
8	Acceptability of the recruitment process
Data collection procedures and outcome measures	
9	Acceptability of assessments and data collection tools
10	Number of questionnaires returned
11	Completion of outcome measures
Intervention acceptability, preliminary responses to the intervention and study procedures	
12	Adherence and reasons for non-adherence including barriers and facilitators to intervention use informed by CFIR
13	Feasibility and acceptability of the intervention content and delivery
14	Evaluation of health economic analysis process
15	Feasibility of fidelity assessment
16	Resource use related to intervention delivery such as training

The recruitment process in Fig. 1 will be followed; however there may be variation in how the process is conducted, particularly due to the variation in types of organisation and professionals involved in the recruitment process. We will therefore capture recruitment strategies, capabilities and acceptability during staff interviews and through recorded information about the steps sites take in recruitment. We will record numbers screened, eligible, consented, dropout, reasons for non-recruitment and attrition from sites. Demographic information of the recruited sample will be collated through baseline questionnaires.

Data collection procedures outlined above will be assessed through monitoring the number of questionnaires returned, and completion of outcome measures. The tools will be tested during the feasibility study to ensure their reliability and acceptability in capturing the required data. PPI representatives will advise on these tools, to refine and provide feedback, ensuring the data collection tools are patient-centred and practical. This aligns with the study’s overarching emphasis on engaging PPI throughout all research phases. In the process, we will minimise burden on the participant through support and allowing for adequate breaks when completing questionnaires. The participant’s ease of completion will be recorded.

An assessment of fidelity to the intervention will be conducted to inform the feasibility of intervention implementation and to assess the feasibility of fidelity assessment procedures. Facilitators will be asked to complete a checklist reporting intervention use, and a post intervention questionnaire about their own experiences of delivering the intervention. People with dementia and family carers will complete a post intervention questionnaire self-reporting intervention use. Interviews will explore barriers and facilitators to intervention use, and feasibility and acceptability of intervention content and delivery. A professional from each site will complete a PCAT prior to intervention delivery to consider potential barriers and facilitators [54]. The health economic evaluation will adopt a societal perspective, incorporating both health and social care sector costs and broader societal costs [61]. This approach recognises that medicines self-management interventions may have implications beyond the healthcare system. Data collection tools will be designed to capture costs and resource use comprehensively, drawing on existing literature in dementia care and medicines management. The resources required for the development and delivery of the intervention, including training, professional time, and materials, will be documented. These data will be obtained from participant and informal carer reports, and detailed tracking of intervention activities.

### Criterion for progression to a definitive randomised controlled trial

Criteria to determine if the intervention could proceed to a randomised controlled trial are identified below. We will apply ‘Red’, ‘Amber’, and ‘Green’ (RAG) criteria, an established method for trials [62], which we have previously applied [63]. Key criteria will be based on study recruitment, intervention delivery, and participant attendance as outlined in Table 3. Whilst participants will be screened as part of the recruitment process, for the purposes of RAG criteria, the recruitment time defined does not include time for screening participants, as advised by statisticians at a clinical trials unit. Should some of the metrics not be in green, which would require a minimum of 50% Recruitment, ‘19% Delivery of the Managing My Medicines’ Guide’, ‘19% Follow up visits’ and ‘19% Evidence of the feasibility to collect outcomes measures’, we will consider how and if we can continue to proceed to trial with modifications. The results will be discussed within the research team and with the project steering committee to determine next steps.

**Table 3:** Progression criteria

Decision Criterion	Go (Green) Proceed with no modifications	Modify (Amber) Continue—with modifications	Stop (Red) Do not proceed to RCT
Target Recruitment			
72 participants in 6 weeks. 12 participants across all sites per week Assessed 6 weeks after starting feasibility recruitment in each site	3–4 (participants per site per week)	2 (participants per site per week)	< 2 (participants per site per week)
Delivery of the Managing My Medicines’ Guide			
Professional reporting delivering the Managing My Medicines’ Guide, and person with dementia/family carer receiving it	54–72	26–53	0–25
Follow-up visits			
Professional reported evidence of follow up visit/call carried out and person with dementia/family carer receiving it	54–72	26–53	0–25
Evidence of the feasibility to collect outcome measures			
Completed measures	54–72	26–53	0–25

We will test the feasibility of collecting outcome data through tools outlined in Table 4.

**Table 4:** Outcome measures

Outcomes measures for person with dementia	
Baseline, end month 1, end month 2, post intervention	
Medication Understanding and Use Self-Efficacy Scale (MUSE) [68]	The Medication Understanding and Use Self-Efficacy Scale measures the efficacy of understanding and using prescription medication. This valid and reliable tool measures learning about medications and adherence and can be used with participants with different literacy levels [68]
Healthcare resource use questionnaire	Use of health services, related travel and support from family and friends
EQ-5D-5L [64]	EuroQol 5 Domains 5 Level [64]
DEMQOL [69]	Condition-specific measure of health-related quality of life in dementia [69]
Generalised self-efficacy scale [70]	The General Self-Efficacy Scale is a 10-item scale measuring perceived self-efficacy such as ability to cope with difficult tasks [70]
Medicines information	Medicines information such as drug name, delivery and duration
Post intervention	
Patient experience questionnaire self-reporting intervention use	Skills and knowledge related confidence levels
Outcome measures for family carers	
Baseline, end month 1, end month 2, post intervention	
Healthcare resource use questionnaire	Use of health services, related travel and support from family and friends
EQ-5D-5L [64]	EuroQol 5 Domains 5 Level
EQ- 5D-5L-Proxy [64]	EQ-5D proxy is an instrument evaluating the proxy’s perception of health related quality of life with one question for mobility, self-care, usual activities, pain/discomfort, and anxiety/depression [64]
DEMQOL Proxy V4 [69]	DEMQOL involves cost-effectiveness analysis using quality-adjusted life-years as the measure of effectiveness for people living with dementia [69]
Generalised self-efficacy scale [70]	The General Self-Efficacy Scale is a 10-item scale measuring perceived self-efficacy such as ability to cope with difficult tasks [70]
Carer experience scale [67]	To record the caring experience for use in economic evaluation [67]
Post intervention	
A carer experience questionnaire self-reporting intervention use	Skills and knowledge related confidence levels

Healthcare resource use will include GP visits, nurse consultations, hospitalisations, and social care services. Out-of-pocket expenses incurred by participants and their family carers, such as travel costs and over-the-counter medications, will also be recorded.

In line with NICE recommendations, the EQ-5D-5L will be used to measure health-related quality of life for participants and carers, with additional exploration of measures such as DEMQOL and the Carer Experience Scale (CES) to capture dementia-specific and carer-relevant outcomes [61, 64–67].

### Data analysis

#### Quantitative analysis

Descriptive statistics will be employed to analyse survey data, such as means, median, mode, and average range. We will report on exploratory analysis of cost data. Costs will be estimated for healthcare resource use, productivity losses, and out-of-pocket expenses. The unit costs for health and social care services will be derived from national sources such as the Personal Social Services Research Unit (PSSRU) costs [71], NHS Reference Costs [72], and the British National Formulary (BNF) [73].

#### Qualitative analysis

Qualitative semi-structured interviews with the person living with dementia, family carers, and staff will be analysed to assess barriers and enablers to intervention use. Qualitative data will be analysed using the framework method [74]. Framework analysis is frequently applied in UK health research [75]. The process involves 7 key steps as follows: (1) creating an audio and verbatim transcript, (2) familiarisation through reading the interview transcript, with the interview, (3) coding the transcript by labelling key phrases, (4) co-creating codes to apply to all transcripts called a Framework, (5) applying the analytical framework, indexing using existing coding, (6. charting data into a framework matrix', summarising data from categories into a matrix and (7) interpretation of data. We will use the computer software package NVivo [76] to conduct the framework analysis. The evaluation will be underpinned by the consolidated framework for implementation research (CFIR) [55, 56], social cognitive [57], self-efficacy [58], and resilience theory [35].

#### Data management

All investigators and study site staff will comply with the requirements of the Data Protection Act 1998 and the General Data Protection Regulations with regard to the collection, storage, processing and disclosure of personal information and will uphold the core principles.

Risk of breaching confidentiality and data protection has been identified for this study and will be managed appropriately. Data processing will remove any participant identifiable information immediately following the interview or data extraction. Participants will be allocated a unique, unrelated, numerical identifier or pseudonym for analysis purposes. Paper-based data will be transported by secure post or in a secure bag. Electronic data will be transferred via secure nhs.net email account, or a secure data transfer system Environment for Research or encrypted USB device or Dictaphone or WhatsApp [77] or phone. Any paper-based participant identifying information, e.g. consent forms, will be locked in a filing cabinet. Demographic information will be collated into a password protected Microsoft Excel spreadsheet and paper forms subsequently shredded. Care will be taken so that the combination of incidental details, e.g. locality, age and ethnicity, does not lead to individuals being identifiable. All digital audio recordings, typed field notes, transcribed interviews and electronic extracted data must be password protected and stored securely. Only the project team will be able to access the full anonymised data set. Personal information will be stored electronically for 18 months after the end of the study before it is expunged. This is to allow dissemination of findings. Anonymised data will be stored for five years post project end before it is expunged. In line with the General Data Protection Regulations 2018, all participants will be given written and verbal information detailing how their personal information will be processed.

#### Steering group

A pre-existing research group focusing on safe care at home is the steering group for the MAGNET project. The group comprises health researchers and professionals. The steering group will support qualitative analysis of patient data. The group will also advise on how to keep the project grounded in real experience and ensure it is informed by best practice and research.

#### Public and patient involvement (PPI)

PPI is critical to healthcare research. Our systematic review also identified a lack of PPI in studies to enhance medicines’ self-management for people living with dementia [36]. We therefore organised a MAGNET patient public involvement group. The PPI group will ensure the project is relevant to authentic experience and challenges for people living with dementia and their family carers in safely managing their medicines.

Our PPI group lead is a co-investigator on the project. The group comprises 5 people including a person living with dementia and former family carers of people with dementia. The group is key advisors and contributes to research activities across the duration of the project. Having been involved in the initial project design, the first two phases of the MAGNET programme which involved exploring how people with mild-moderate dementia (with and without their family carers) self-managed their medicines, and co-designed an intervention, the group will continue to advise on phase 3.

Advisory activities will continue to include providing guidance on ethical issues, recruitment, designing project materials, and dissemination. For example, the group advises on how to make project materials more inclusive and accessible. Where possible, data collection tools will be translated, and the researcher will be available to administer the questionnaires when necessary. The PPI group was also involved in the co-design of MAGNET, writing for publication and qualitative data analysis.

#### Ethics

A favourable opinion has been given from East of England–Essex REC (Reference: 24/EE/0164.

This project involves people living with mild to moderate dementia who by nature of this condition may be vulnerable. It is a priority that participation in this project does not cause extra burden or harm. Careful consideration of associated ethical issues has taken place and advice sought from the PPI group and healthcare professionals. The project is non-intrusive, and methods are flexible to meet the needs of participants, e.g. interviews can be completed in place of interviewees' choosing. Participants are free to withdraw from the study at any time. Data collection tools and patient documentation are co-designed with the PPI group, underpinned by Good Clinical Practice [78], to ensure they are appropriate and manageable. For any amendment to the study, the project team, in agreement with the sponsor, will submit information to the appropriate body for them to issue approval for the amendment.

#### Dissemination policy

Our PPI group will inform our strategy, particularly on how we can widen dissemination within communities. We will publish our work in high-quality international academic journals, appropriate professional journals and present at conferences. Social media platforms will be used to further disseminate our work. We will contact organisations relevant to medicines management and dementia to share our findings. We will also relate our findings back to the organisations we have worked with.

#### Authorship eligibility guidelines and any intended use of professional writers

The success of the project depends upon the collaboration of all participants. For this reason, credit for the main results will be given to all those who have collaborated in the project, through authorship and contributorship. Uniform requirements for authorship for manuscripts submitted to medical journals will guide authorship decisions.

A final study report will be developed and agreed upon by all co-applicants for the funder as well as interested organisations and stakeholders.

## Discussion

People living with dementia and family carers need support within the community with self-management of their medicines. This non-randomised feasibility study will assess the MAGNET intervention which aims to do this. Should feasibility be supported, additional funding will be sought for the development and implementation of a randomised controlled trial.

## Data Availability

Data sharing is not applicable to this article as no datasets were generated or analysed during the current study.

## Abbreviations

PPI: Patient Public Involvement
CFIR: Consolidated Framework for Implementation Research [55, 56]
EBCD: Experience based co-design [44]
MUSE: Medication Understanding and Use Self-Efficacy Scale [68]
PCAT: Pragmatic Context Assessment Tool [54]
R & D: Research and Development
